# Enhanced expression of Survivin has distinct roles in adipocyte homeostasis

**DOI:** 10.1038/cddis.2016.439

**Published:** 2017-01-05

**Authors:** Liping Ju, Xiaoyan Zhang, Yujie Deng, Junfeng Han, Jian Yang, Shuqin Chen, Qichen Fang, Ying Yang, Weiping Jia

**Affiliations:** 1 Shanghai Key Laboratory of Diabetes, Shanghai Institute for Diabetes, Shanghai Clinical Medical Centre of Diabetes, Shanghai Key Clinical Centre of Metabolic Diseases, Department of Endocrinology and Metabolism, Shanghai Jiao-Tong University Affiliated Sixth People's Hospital, Shanghai, China; 2 Department of Endocrine and Metabolic Diseases, Institute of Endocrine and Metabolic Diseases, Ruijin Hospital, Shanghai Jiao-Tong University School of Medicine, Shanghai, China; 3 Department of Endcrinology, The Affiliated Hospital of Qingdao University, Qingdao, China

## Abstract

Although precisely controlled lipolysis is crucial for maintaining physiological levels of circulating free fatty acids in response to energetic stress, the underlying mechanisms by which this process is governed remain poorly understood. Survivin is a gene that has been found to be highly expressed in the most common human tumors, and it is considered to be associated with tumorigenesis. Survivin expression in normal tissue is developmentally downregulated and is undetectable in most terminally differentiated adult tissues. Here, we report that Survivin expression in mature adipocytes from murine white adipose tissue can be highly induced under high-fat diet feeding conditions. During the adipocyte differentiation of 3T3-L1 preadipocytes and mesenchymal C3H10T1/2 cells, Survivin expression is gradually decreased and almost undetectable in fully differentiated adipocytes. However, it can be expressed again upon insulin exposure, through the PI3K/mTOR signaling pathway. Nevertheless, Survivin overexpression is sensitive to nutritional deprivation, and expression markedly decreases in response to starvation with Hank's buffered salt solution challenge. The ectopic expression of Survivin downregulates expression of Adrb3 and then decreases the production of cAMP, while Fsp27 protein levels are upregulated as a result of reduced protein degradation. This in turn inhibits isoproterenol-stimulated adipocyte lipolysis. Survivin also attenuates DNA damage related to PARP activation and inhibits TNF*α*-induced lipolysis, suggesting that Survivin may facilitate adipocyte maintenance in response to inflammatory stimuli. Further studies will be undertaken to determine whether Survivin is critical for lipid storage to maintain metabolic homeostasis *in vivo*.

Adipose tissue is the primary fat storage depot and a major source of metabolic fuel, whose dysfunction is closely associated with obesity and related metabolic phenotypes. In response to excess caloric intake, adipose tissue expands to store extra energy through hypertrophy and/or hyperplasia, thereby preventing ectopic lipid deposition and lipotoxicity.^1, 2^ Triacylglycerols within the lipid droplets of adipocytes are excellent stored forms of energy. Under normal physiological conditions, the stored triacylglycerols are hydrolyzed to glycerol, and fatty acids via *β*-adrenergic receptors (*β*-AR) signaling. These receptors in turn activate adenylyl cyclase to generate cAMP. The cAMP-activated protein kinase A directly phosphorylates perilipin and hormone sensitive lipase (HSL), which regulates the lipolytic process in adipocytes.^3^ However, lipolysis can also be triggered by inflammatory signals.^4^

In obese individuals, excessive lipolysis contributes to high circulating levels of fatty acids and causes deleterious effects on insulin signaling in peripheral tissues. Therefore, slowing down free fatty acid turnover in these individuals may improve insulin resistance. An important signaling pathway involved in inhibiting lipolysis is the mammalian target of rapamycin (mTOR) pathway. Overnutrition causes hyper-activation of mTOR in adipocytes to maintain low circulating levels of free fatty acids, primarily by antilipolytic action, which preserves normal lipid partitioning and overall metabolic fitness. Circulating insulin plays a key role in this process. The failure of insulin to restrain lipolysis contributes to abnormal lipid partitioning and metabolic disease.^5, 6, 7^

Survivin is a member of the inhibitor of apoptosis family that has been found to abundantly expressed in fetal tissues and the most common human tumors, and was considered absent in normal tissues in initial studies.^8^ However, growing evidence indicates that Survivin expression in normal tissue is developmentally downregulated and is low in most terminally differentiated adult tissues.^9, 10^ Survivin can also be upregulated in several normal adult tissues under certain pathological conditions, such as hypoxia^11^ and inflammation.^12^ Moreover, terminally differentiated neutrophils are able to induce the Survivin gene expression in response to granulocyte/macrophage colony-stimulating factor or granulocyte colony-stimulating facto stimulation. Elevated Survivin levels in mature neutrophils are also observed in patients with distinct inflammatory diseases.^13^

Survivin has been reported to involved in several crucial cell processes, such as apoptosis and cell division, and has received considerable attention since its discovery because of its likely role in tumorigenesis.^14, 15^ However, subsequent studies revealed that Survivin is essential for steady-state hematopoiesis^9^ and has critical roles in T cells's undergo impaired development, maturation and homeostasis.^10^ Overexpression of Survivin in mature neutrophils is required to inhibit apoptosis in a cell cycle–independent manner under inflammatory conditions, indicating that Survivin may also have an important role in terminally differentiated cells.^13^ However, no reference is available regarding the expression and potential function for Survivin in adipocytes.

Here, we show that Survivin expression in white adipose tissue is low but can be highly induced in response to a high-fat diet (HFD). Subfractionation of adipose depots revealed that Survivin was mainly expressed in the adipocyte fraction rather than in the stromal vascular fraction (SVF) of the epididymal fat, and to a lesser extent in the adipocyte fraction of the subcutaneous fat. During adipocyte differentiation, Survivin expression is gradually decreased and almost undetectable in fully differentiated adipocytes. However, Survivin expression increases upon insulin exposure through phosphatidylinositol 3-kinase (PI3K)/mTOR signaling. The ectopic expression of Survivin in 3T3-L1 cells does not affect adipocyte differentiation, but rather inhibits isoproterenol-stimulated adipocyte lipolysis via the *β*-Adrenergic/cAMP pathway. Survivin also attenuates DNA damage-related stress responses, as well as TNF*α*-induced lipolysis, suggesting that Survivin may facilitate adipocyte maintenance in response to inflammatory stimuli. Taken together, the current findings identify a previously unknown role for Survivin as a key regulator of adipocyte metabolism.

## Results

### Enhanced expression of Survivin in the adipocyte fraction from the white adipose tissue of HFD-fed mice

**Figure 1:**
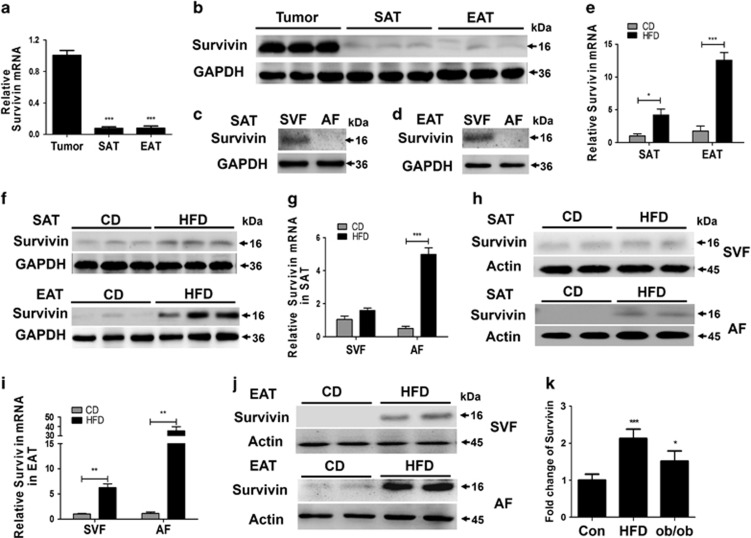
Survivin is induced in mature adipocytes from murine white adipose tissue by HFD feeding. Survivin expression in the subcutaneous and epididymal fat of 8-week-old mice was determined by qRT-PCR (**a**) and western blot (**b**); lung tumor tissues from nude mice used as a positive control. The SVF and adipocyte fraction (AF) were divided from the subcutaneous adipose tissue (SAT) and epididymal adipose tissue (EAT) of 8-week-old mice, then protein levels of Survivin in the SVF and adipocyte fraction divided from SAT (**c**) and EAT (**d**) were respectively determined. (**e**–**j**) C57BL/6 mice were fed with HFD or standard chow diet (CD) for 24 weeks; expression levels of Survivin in SAT and EAT were determined by qRT-PCR (**e**) and western blot (**f**). The SVF and adipocyte fractions were divided from SAT and EAT respectively, then the mRNA and protein content of Survivin in the SVF and adipocyte fraction divided from SAT (**g** and **h**) and EAT (**i** and **j**) was determined, respectively. (**k**) Microarray analysis of GSE27017, relative expression of Survivin in 6 months HFD and 6 months old ob/ob mice. *P<0.05, **P<0.01, ***P<0.001

Gene and protein expression analyses of Survivin were first performed in subcutaneous and epididymis fat from 8-week-old chow-fed C57BL/6 mice and in lung tumor tissues from nude mice (positive control). As predicted, Survivin has a high expression in tumor tissues. Comparatively, the levels of Survivin are low, yet detectable in normal subcutaneous and epididymis fat (Figures 1a and b). We further separated the SVF and adipocyte fractions from the subcutaneous fat (Figure 1c) and epididymal fat (Figure 1d) and found that Survivin was predominantly expressed in the SVF (Figures 1c and d).

To determine the changes in Survivin expression under obese conditions, we first assessed the mRNA and protein levels of Survivin in adipose tissue from male C57BL/6 mice fed standard chow or a HFD for 24 weeks. Compared with control mice, obese mice showed a nearly sevenfold increase in Survivin mRNA in the epididymal fat, while expression in the subcutaneous fat was nearly fourfold upregulated (Figure 1e). Consistent with the mRNA results, the Survivin protein content was significantly increased in the epididymal fat and slightly increased in the subcutaneous fat of obese mice (Figure 1f). Subfractionation of adipose depots revealed that in the subcutaneous fat of HFD-fed mice, Survivin mRNA expression was upregulated nearly fivefold in the adipocyte fraction but unchanged in the SVF (Figure 1g). Furthermore, Survivin was elevated nearly 32-fold in the adipocyte fraction and nearly sixfold in the SVF from epididymal fat (Figure 1i).There were consistent changes in Survivin protein levels in the SVF and adipocyte fractions from both subcutaneous fat (Figure 1h) and epididymal fat (Figure 1j).

Moreover, microarray analysis of GSE27017 supports our results that Survivin expression in epididymal adipocytes is upregulated in both HFD-fed (for 12 weeks) mice and in ob/ob mice (Figure 1k).

### Enhanced expression of Survivin in fully differentiated adipocytes via PI3K/mTOR signaling upon insulin exposure

**Figure 2:**
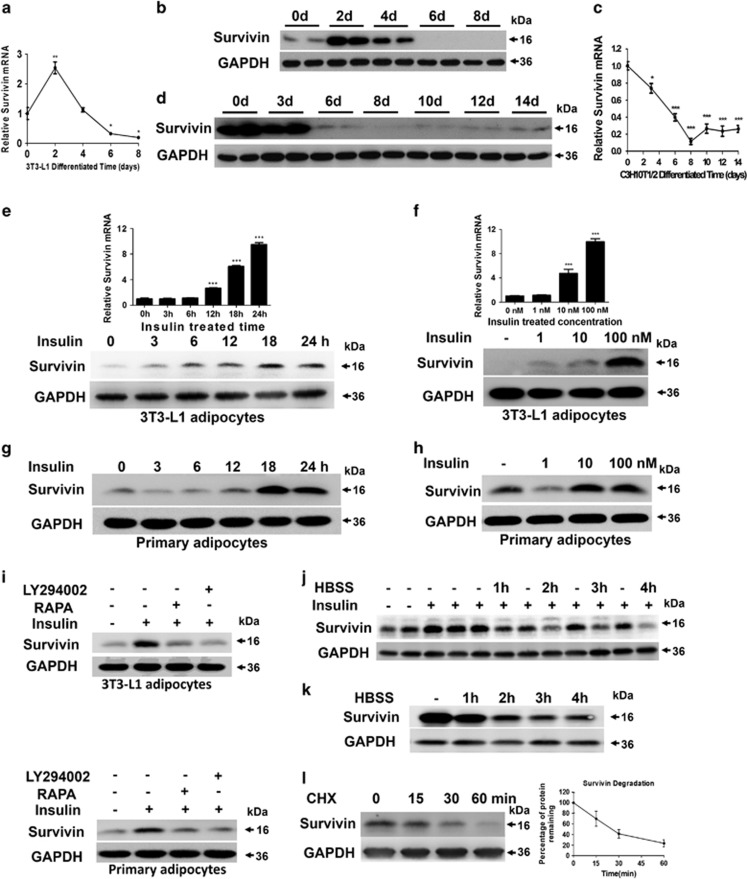
Survivin is expressed upon insulin exposure through the PI3K/mTOR pathway in mature adipocytes. During the differentiation process of 3T3-L1 cells and C3H10T1/2 cells, Survivin mRNA and protein levels were determined by qRT-PCR (**a** and **c**) and western blot (**b** and **d**). Survivin expression was detected by qRT-PCR and western blot in 3T3-L1 adipocytes, which were treated with 100 nM insulin for the indicated time (**e**) and insulin at the indicated concentrations for 24 h (**f**). Primary adipocytes, isolated and cultured from the SVF of subcutaneous fat, were induced to differentiate into mature adipocytes. Then Survivin content was determined by western blot after treating with 100 nM insulin for the indicated time (**g**) and insulin at the indicated concentrations for 24 h (**h**). Survivin content was immunobloted after rapamycin (10 nM) and LY294002 (50 *μ*M) for one hour prior to insulin exposure (100 nM) both in 3T3-L1 adipocytes and primary adipocytes (**i**). (**j**) 3T3-L1 adipocytes were incubated with insulin (100 nM) for 24 h, then incubated with or without HBSS for the indicated time. Immunoblots determined the levels of Survivin and GAPDH. (**k**) Protein levels of Survivin were detected by western blot in 3T3-L1 adipocyte overexpressing Survivin after incubation with HBSS for the indicated time. (**l**) The stability of ectopic expression of Survivin was evaluated in a cycloheximide (CHX, 100 *μ*g/ml) chasing experiment. Cells were harvested 0, 15, 30 or 60 min after the addition of CHX. The ectopic expression of Survivin protein level was evaluated using western blot. *P<0.05, **P<0.01, ***P<0.001

A HFD induced the expression of Survivin in adipose tissue, mainly in the adipocyte fraction. Furthermore, in various cancer cells, Survivin is critically regulated by some cell signaling molecules, such as HIF-1*α*, PI3K/AKT, mTOR and AMPK.^16, 17, 18^ Therefore, we hypothesized that the expression of Survivin is sensitive to nutritional status and is regulated by nutrient-sensing pathways in adipocytes. To test this possibility, we first respectively examined the expression of Survivin in the differentiation process of 3T3-L1 preadipocytes and mesenchymal C3H10T1/2 cells. In 3T3-L1 cells, both mRNA and protein levels of Survivin were transiently increased on day 2, then dramatically decreased and were almost undetectable in fully differentiated adipocytes (Figures 2a and b). The differentiation of pluripotent C3H10T1/2 stem cells involves two distinct stages: commitment to preadipocytes and the subsequent development of adipocytes. Survivin was highly expressed in the pluripotent stem cells stage (before day 3) and dramatically decreased to undetectable levels during the process of terminal differentiation (Figures 2c and d).

Nutrient excess causes hyper-activation of mTOR in adipocytes. To confirm whether Survivin was regulated by the mTOR pathway, insulin was used to active mTOR signaling in 3T3-L1 adipocytes. After insulin treatment, expression of Survivin increased in a time-dependent and dose-dependent manner in 3T3-L1 adipocytes (Figures 2e and f). We further isolated the primary SVF fraction from the subcutaneous fat of C57BL/6 mice and induced adipocyte differentiation *in vitro* and also found that the protein level of Survivin increased following exposure to insulin (Figures 2g and h). Furthermore, 3T3-L1 adipocytes and SVF-induced adipocytes were stimulated with insulin in the presence or absence of rapamycin and LY294002, which are inhibitors of the mTOR and PI3K, respectively. The results indicated that although stimulation with insulin promoted a robust increase in Survivin in both 3T3-L1 adipocytes and primary adipocytes, this effect was almost completely blocked by rapamycin and LY294002 (Figure 2i), suggesting that the expression of Survivin might be regulated by the PI3K/mTOR pathway.

Here, 3T3-L1 adipocytes were starved in Hank's balanced salt solution (HBSS) for a period of time after a 24 h insulin treatment. Insulin upregulated the expression of Survivin, which was then obviously downregulated by 4 h of exposure to HBSS (Figure 2j), suggesting that insulin-induced Survivin overexpression is sensitive to nutrient deprivation. In addition, ectopic expression of Survivin via lentiviral gene transfection is also sensitive to HBSS treatment (Figure 2k) and has a short lifetime (Figure 2l).

### DEGs identified between control and Survivin overexpression groups in adipocytes

We next assessed the underlying function of Survivin in adipocytes by lentiviral vector-mediated Survivin overexpression in 3T3-L1 cells, and then differentiated these into mature adipocytes. Morphological differentiation, as well as cellular triglyceride assays on day 8 of differentiation showed that both control and Survivin overexpressing 3T3-L1 cells were able to differentiate into adipocytes to a similar extent (Supplementary Figures 1A and C), which was further confirmed by the absence of significant differences in the expression of PPAR*γ* and C/EBP*α* between these two groups (Supplementary Figure 1B).

**Figure 3:**
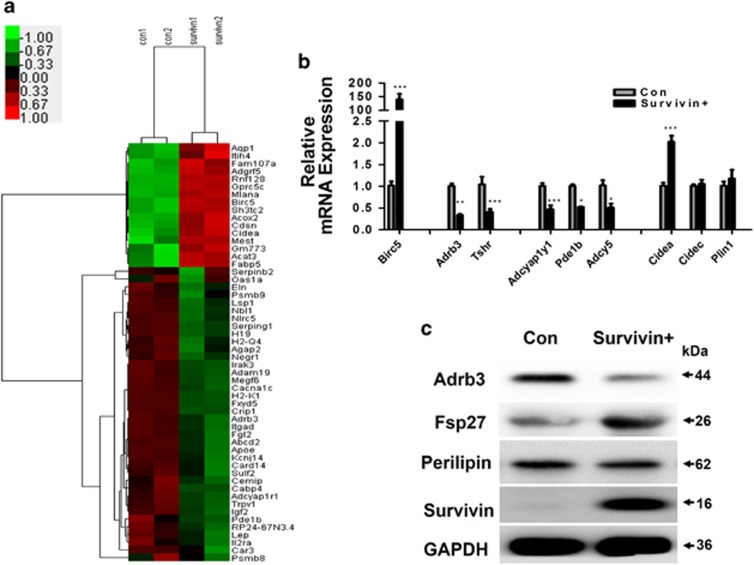
RNA sequencing analysis of Survivin's potential function in adipocytes. RNA sequencing and data analysis of the control and Survivin overexpression group. cDNA libraries were constructed from total RNAs extracted and analyzed sequences on the Illumina HiSeqTM 2500. (**a**) Heatmap analysis displayed the DEGs between the control and Survivin overexpression group. A red–green color scale was used to reflect standardized gene expression, with red representing a high expression and green representing a low expression (scale shown in the upper left). Cutoff values were FDR<0.05 and a fold change ratio>1.5 change. Results of RNA sequencing were tested in 3T3-L1 adipocytes (**b** and **c**). Lipolysis-associated protein, cAMP metabolism related genes and lipid droplet envelope protein were tested by qRT-PCR (**b**). (**c**) Protein levels of Adrb3, Fsp27 (Cidec), Perilipin, Survivin and GAPDH were determined by western blot. *P<0.05, **P<0.01, ***P<0.001

To identify whether Survivin has a role in mature adipocytes, RNA sequencing analysis of differentially expressed genes (DEGs) (FDR<0.05, Fold change>1.5) was used to compare the Survivin overexpression with the control group. Ultimately, 54 significantly differentially genes were identified (Supplementary Data File 1), including 16 upregulated and 38 downregulated genes. Among these genes, lipolysis-associated protein Adrb3 and Tshr were significantly decreased, while lipid droplet envelope protein Cidea was significantly increased. Consistently, expression of cAMP metabolism-associated genes, Adcyap1r1 and Pde1b, were also overtly decreased (Figure 3a). To confirm the differential expression of genes identified by RNA sequencing, we chose eight candidate genes: Adrb3, Tshr, Adcyap1r1, Pde1b, Adcy5, Cidea, Cidec (Fsp27) and Plin1 (perilipin), whose expression was validated by qRT-PCR. We found that the expression of these genes was consistent with the RNA sequencing data (Figure 3b). At the protein level, Fsp27 and perilipin are mainly expressed in white adipocytes, so we further compared Adrb3, as well as the Fsp27 and perilipin protein contents between the control and Survivin overexpression group. We found that Adrb3 was markedly downregulated, while Fsp27 was significantly upregulated in the Survivin overexpression group, suggesting that Survivin may have a role in the lipolysis process in adipocytes.

RNA sequencing analyses also revealed that Survivin overexpression did not alter the gene expression patterns associated with adipocyte differentiation, fatty acid *β*-oxidation, lipogenesis and lipase (Supplementary Figure 1D). Furthermore, qRT-PCR results from 3T3-L1 adipocytes showed that adipocyte differentiation-related gene expression was consistent with the RNA sequencing data (Supplementary Figure 1E).

### Survivin inhibits lipolysis via downregulated expression of Adrb3 and its corresponding pathway, as well as upregulated Fsp27 expression

**Figure 4:**
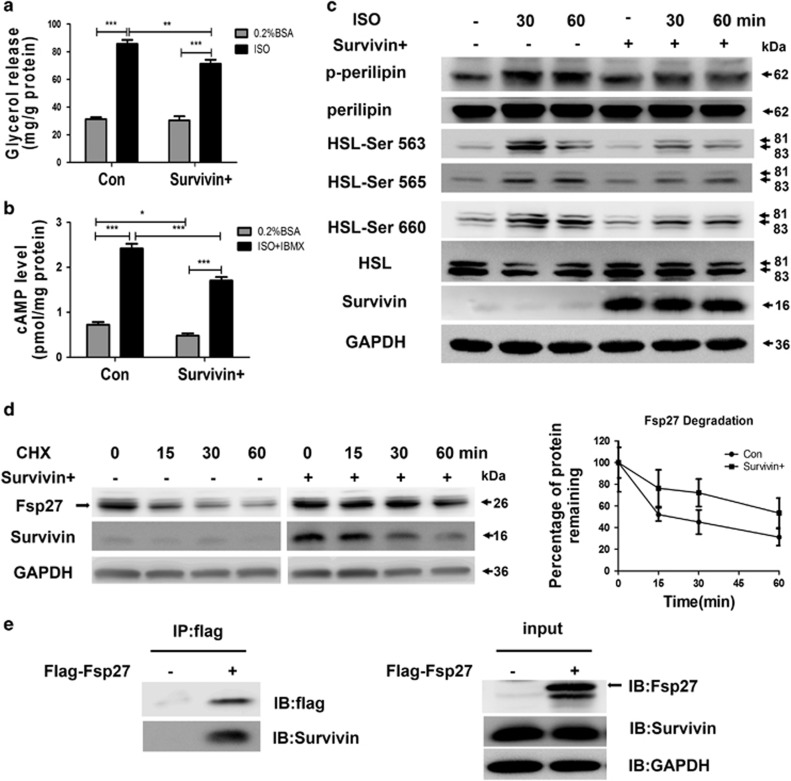
Ectopic expression of Survivin inhibits lipolysis by targeting Adrb3 and Fsp27. (**a**) 3T3-L1 adipocytes were stimulated with 1 *μ*M isoproterenol (ISO) for 3 h, then media was removed for glycerol measurement. (**b**) 3T3-L1 adipocytes were stimulated with 1 *μ*M ISO for 1 h and also treated with 200 *μ*M IBMX along with ISO stimulation, then cells were extracted for cAMP assay. (**c**) 3T3-L1 adipocytes were stimulated with 1 *μ*M ISO for indicated time. Total proteins were subjected to SDS-PAGE. Immunoblots for GAPDH, phospho-HSL at Ser-563, Ser-565, and Ser-660, phospho-perilipin using phospho (Ser/Thr)-PKA substrate antibody to detect protein at 62 kDa and total HSL and perilipin were performed on cell lysates. (**d**) Fsp27 stability was evaluated in a cycloheximide (CHX, 100 *μ*g/ml) chasing experiment. Immunoblot of Fsp27 after 15, 30 and 60 min treatments with CHX in 3T3-L1 adipocytes. ‘Black circles'=control group; ‘black squares'=Survivin overexpression group. (**e**) 293T cells were infected with Birc5 lentivirus to overexpress Survivin, then transfected with flag-tagged Fsp27, as indicated, and the lysates were immunoprecipitated with flag beads. *P<0.05, **P<0.01, ***P<0.001

Given that lipolysis-associated protein Adrb3, as well as cAMP metabolism-associated genes, Adcyap1r1 and Pde1b, were significantly downregulated and the lipid droplet envelope protein Fsp27 was markedly upregulated in the Survivin overexpression group, we hypothesized that Survivin could influence lipolysis via the cAMP pathway and influence the stability of adipocytes via the lipid droplet envelope protein Fsp27. To investigate this possibility, we first tested whether Survivin overexpression could attenuate the action of isoproterenol on lipolysis. 3T3-L1 adipocytes were treated with 1 *μ*M isoprenaline and glycerol content in the incubation medium was determined as a measure of lipolysis. Survivin did not affect basal lipolysis significantly, but it robustly decreased isoprenaline-induced lipolysis (Figure 4a). In addition, isoproterenol treatment strongly elevated cAMP levels, and overexpression of Survivin markedly decreased isoproterenol-potentiated cAMP production (Figure 4b). Consistent with this result, Survivin also dramatically inhibited HSL Ser-563, Ser-565 and Ser-660 phosphorylation, as well as perilipin phosphorylation, induced by isoproterenol (Figure 4c). Given that Survivin increased Fsp27 protein content without altering its mRNA levels, we speculate Survivin may have an effect on the stability of the Fsp27 protein, resulting in higher expression levels. The stability of the Fsp27 protein was estimated by measuring these protein levels after treatment with 100 *μ*g/ml cycloheximide. As expected, we observed that overexpression of Survivin delayed degradation of Fsp27, increasing its half-life from about 15–30 min to >1 h (Figure 4d). As Survivin inhibited the degradation of Fsp27, we postulated that Survivin and Fsp27 might interact with each other. Indeed, Co-IP assays in 293T cells demonstrated that Survivin can interact with Fsp27 (Figure 4e).

### Survivin partly rescues TNF*α*-induced changes in gene expression involved in DNA damage repair and lipolysis

To explore the potential role of Survivin in obesity-related inflammation stress, we performed RNA sequencing assays to detect the mRNA expression differences under Survivin overexpression in response TNF*α* challenge. In series cluster analysis, we identified eight possible profiles (Supplementary Figure 2A), which represent the overall expression patterns. Of these, profile 5 (point 1<point 2>point 3) and profile 2 (point 1>point 2<point 3) contained 330 DEGs and 75 DEGs, respectively. These two profiles suggest Survivin may be able to rescue against TNF*α* treatment.

Then, we merged these two profiles (Supplementary Data File 2) to identify potential biological processes. Enriched Gene Ontology (GO) terms are shown in Supplementary Figure 2B and arranged according to biological processes. This analysis revealed that the most enriched biological processes were mainly related to cell cycle, mitotic nuclear division, cell division, chromosome segregation and DNA repair.

Subsequently, we built the GO tree to perform deep analysis through analyzing the interactions among the significant GO terms (*P*-value<0.01). Supplementary Figure 2C suggested that DNA metabolic processes, chromosome segregation, and the mitotic cell cycle are the key biological events affected by Survivin overexpression in response to TNF*α* challenge. Moreover, DNA replication and DNA repair are the dominant elements in the DNA metabolic process.

**Figure 5:**
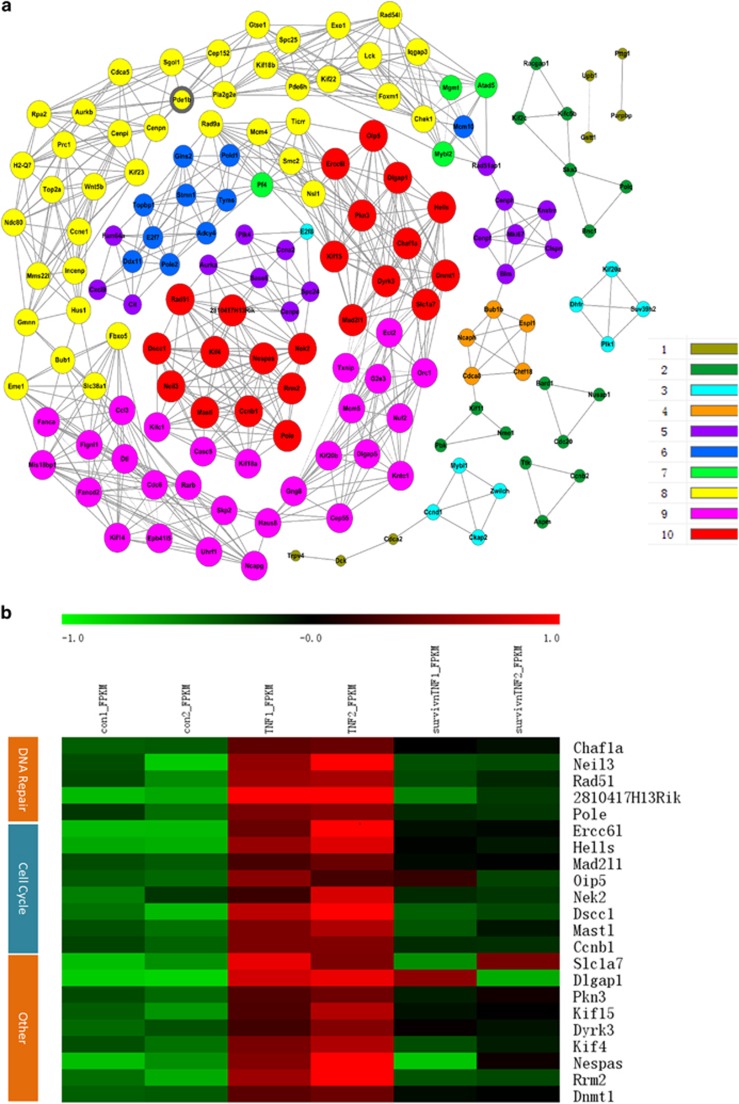

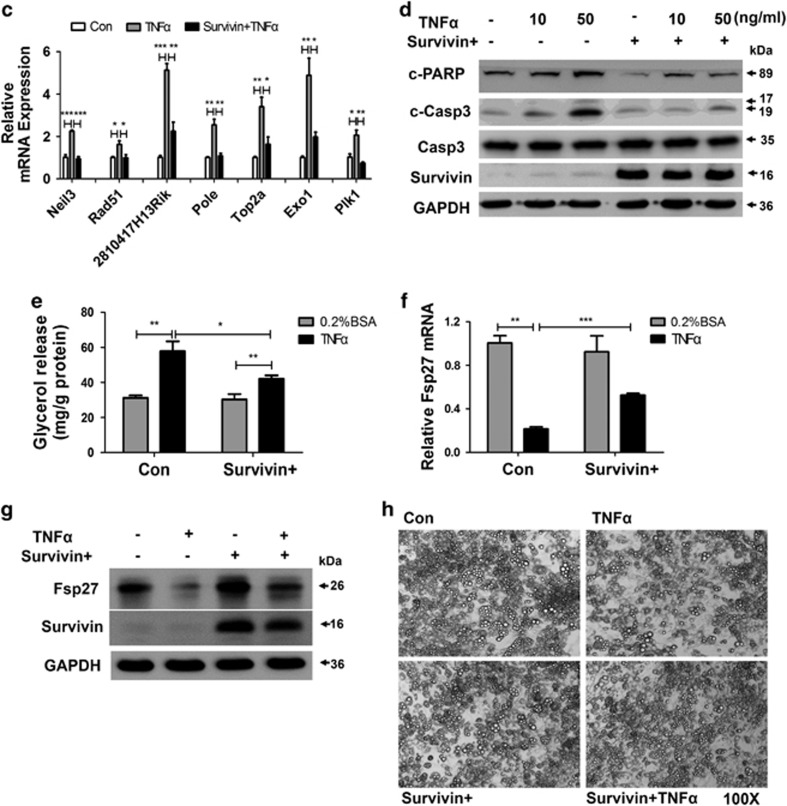
Survivin partly rescues TNF*α*-induced changes in gene expression involved in DNA repair pathways and related PARP activation, as well as lipolysis. The mRNA expression differences were detected by RNA sequencing analysis, and series cluster analysis was introduced to discover the expression trend of DEGs from the control, TNF*α*, and Survivin overexpression+TNF*α* group. Two strains of series cluster analysis were merged, and DEGs were used to do a deep analysis (**a** and **b**). (**a**) Co-expressed genes and their networks in DEGs. A k-core of a given gene indicates its hub status with connection to ‘k' other genes in a network (scale shown in the lower right). (**b**) Heatmap of hub genes with the highest k-core (k-core=10) in Co-expression analysis. Red represents a higher level of gene expression, and green represents a lower level gene expression (scale shown in the upper). FDR<0.05 and a fold change ratio>1.5 change were identified as the boundary value. (**c**) Genes related to DNA repair were evaluated by qRT-PCR. (**d**) Protein levels of PARP and caspase 3 were detected by western Blot. (**e**) 3T3-L1 adipocytes were stimulated with 10 ng/ml TNF*α* for 3 h, then media was removed for glycerol measurement. Fsp27 was tested by qRT-PCR (**f**) and western blot (**g**) in 3T3-L1 adipocytes after stimulation with TNF*α* for 24 h. (**h**) Cell morphology was observed by light microscopy after 6 days of 10 ng/ml TNF*α* treatment. Original magnification, × 100. *P<0.05, **P<0.01, ***P<0.001

We used the DEGs in profile 5 and 2 to construct a gene co-expression network (Figure 5a). Thus, 156 genes in these two profiles were chosen as ‘key regulatory' genes (Supplementary Data File 3). Then, 22 genes with the highest k-core scores were selected to make a comparison with GO terms, and we found that their functions were largely involved in DNA repair and cell cycle (Figure 5b). Compared with the control group, a cluster of genes involved in DNA repair (Chafla, Neil3, Rad51, 2810417H13Rik and Pole) were found to be significantly upregulated in the TNF*α*-treated group, which indicated a cellular stress response upon DNA damage. However, Survivin overexpression robustly downregulated a number of TNF*α*-induced DNA repair genes. The transcript levels of 7 genes (Neil3, Rad51, 2810417H13Rik, Pole, Top2a, Exo1 and Plk1) were re-evaluated using qRT-PCR analysis (Figure 5c). This data agrees with the previous findings that Survivin has a role in the DNA damage repair process in many cancer cells.^13^ Accordingly, TNF*α*-induced cleavage of PARP and caspase 3 activation after DNA damage, an early event in apoptosis, was also largely inhibited by Survivin overexpression (Figure 5d).

Given that Survivin elevated Fsp27 content (Figure 3c) and was involved in the lipolysis of adipocytes (Figure 4a), we explored the potential ability of Survivin to regulate TNF*α*-induced lipolysis, and found that overexpression of Survivin blocked the increase in lipolysis caused by TNF*α* (Figure 5e). Furthermore, results from qRT-PCR analysis and western blots (Figures 5f and g) indicated that Survivin partly reversed the decreases in Fsp27 induced by TNF*α* treatment. Moreover, cell morphology, observed after 6 days of TNF*α* treatment in fully differentiated adipocytes, showed that chronic TNF*α* treatment resulted in an impairment of lipid droplet maintenance in adipocytes. However, Survivin overexpression could effectively reverse this effect (Figure 5h). These results suggested that Survivin might be required for adipocyte survival, as well as the maintenance of adipocyte identity and function upon inflammation tress.

## Discussion

In response to changes in nutritional conditions, precisely controlled lipolysis is crucial for maintaining physiological levels of circulating free fatty acids to maintain normal lipid partitioning and metabolic fitness. It is therefore essential to characterize the mechanism of lipolysis in white adipocytes. We have now shown that Survivin is expressed both in separated murine white adipocytes upon high-fat feeding and in differentiated adipocytes after incubation with insulin. Overexpression studies have revealed that Survivin can inhibit lipolysis through a cAMP-dependent pathway. Moreover, this study has also implicated Survivin as a critical protective factor against TNF*α*-induced adipocyte damage and lipolysis.

The SVF contains a heterogeneous cell population, including endothelial cells, smooth muscle cells, multipotent mesenchymal stem cells and fibroblasts, some of which are undifferentiated and highly proliferating cells.^19^ This may explain why Survivin is readily detectable in cells from the SVF. Mature adipocytes comprise the highest percentage of cells and represent the terminally differentiated cells in adipose tissue. Therefore, Survivin is generally undetectable in this fraction, even though its mRNA is present in amounts almost similar to those in the SVF. However, we determined that Survivin can be expressed in the white adipocytes isolated from HFD-fed mice. To our knowledge, only one study has detected Survivin expression in mature terminally differentiated cells, where high levels of expression were induced in neutrophils upon growth factor stimulation *in vitro* and under inflammatory conditions *in vivo*.^13^ Moreover, we further identified insulin-activated PI3K/mTOR signaling as an important pathway of Survivin up-regulation in fully differentiated adipocytes. Nevertheless, Survivin expression is sensitive to nutrient deprivation and dramatically decreases in response to HBSS challenge. A number of studies have indicated that overfeeding cause hyper-activation of mTOR, thus stimulating adipose tissue expansion by promoting adipogenesis and lipogenesis, as well as inhibiting lipolysis. On the other hand, nutrient deprivation causes de-activation of mTOR, which then reduces lipid storage and the release fatty acids to supply energy.^20^ We speculate that Survivin may be a nutrient sensitive molecule, which acts as a crucial checkpoint in response to mTOR activation. Up-regulation of Survivin may represent a protective mechanism to avoid ectopic lipid deposition and metabolic dysfunction under overnutrition conditions.

Our study demonstrates that overexpression of Survivin downregulates expression of Adrb3 receptors and decreases the production of cAMP. The *β*3-A receptor is predominantly expressed in white and brown adipocytes, which mobilize stored fat and stimulate brown adipose tissue thermogenesis in response to *β*-agonists.^21^ A previous study showed a dramatic reduction in *β*3-AR levels during the fasted/fed transition and hyperinsulinemic-euglycemic clamping in rat adipose tissue,^22^ which occurred together with transcriptional inhibition of the *β*3-AR gene by insulin in adipocytes, suggesting a causal relationship between insulin and *β*3-AR, secondary to food intake. It has also been reported that thiazolidinediones rapidly and reversibly inhibit *β*3-AR mRNA transcription both in brown and white adipose cell lines through PPAR*γ*.^23^ These changes in mRNA levels closely parallel changes in *β*3-AR–mediated responses. In the current study, Survivin was identified as having an inhibitory role in *β*3-AR gene expression, suggesting that the concerted actions of Survivin, insulin, and PPAR*γ* serve to enhance lipid mobilization within adipose tissue under energetic stress conditions. Indeed, we noted that Survivin partly inhibits isoproterenol-induced lipolysis via the impairment of *β*3-AR/cAMP-mediated HSL phosphorylation. Furthermore, we observed an increase in Fsp27 protein levels, mediated by Survivin, which occurred as a result of reduced protein degradation. Since Fsp27 is a lipid droplet-associated protein that enhances the formation of enlarged lipids and promotes triglyceride accumulation within lipid droplets,^24^ this data shows that Survivin has lipogenic effects.

Survivin was initially characterized as the most powerful inhibitor of apoptosis, which has key roles in the suppression of apoptosis and stimulation of DNA repair, thus promoting cancer cell survival under various stresses.^25, 26^ It was reported that Survivin decreases radiation-induced DNA double-strand breaks and promotes DNA repair in several cancer cells.^27^ As DNA damage during early apoptosis can be repaired via defense mechanisms against the apoptotic cell response,^28^ we speculate that Survivin may also play an essential role in the regulation of adipocyte survival under certain conditions. Expectedly, Survivin dramatically downregulates a cluster of TNF*α*-induced DNA repair-related genes, and inhibits DNA damage-related PARP activation. Rodent studies found that adipocytes may die by necrotic and apoptotic mechanisms and showed an increase in the death rate in obesity.^29, 30^ Efficient DNA repair is needed in terminally differentiated cells with a long life span. HFD-induced DNA damage is a key cause of adipocyte senescence and death, which is critically involved in adipose tissue inflammation and metabolic abnormalities.^31^ A mouse model with a targeted deletion of the DNA repair gene, Ercc1, in adipose tissue reveals fat depot loss and systemic insulin resistance.^32^ However, the roles of molecules related to DNA repair in adipocytes still remain largely unexplored. Data presented here suggest that Survivin-mediated DNA repair mechanisms may facilitate adipocyte maintenance in response to inflammatory stress.

There are several limitations of the current study. First, the mechanisms that regulate Survivin mRNA and protein expression in adipocytes are largely unclear. It is also possible that other hormones and nutrients are involved in this process. Secondly, there is insuffient *in vivo* evidence to investigate the role of Survivin in the maintenance of energy homeostasis. Generating adipose-specific Survivin knockout mice is required for future studies.

This study demonstrates that Survivin can be expressed upon insulin stimulation *in vitro* and under HFD-feeding conditions *in vivo*. Overexpressed Survivin inhibits lipolysis through Adrb3/cAMP signaling and attenuates TNF*α*-induced DNA damage-related stress responses. Accordingly, further studies will be undertaken to investigate whether Survivin is critical for lipid storage to maintain metabolic homeostasis. If so, it will expand our understanding of the biological processes involved in lipid storage under overnutrition conditions.

## Materials and Methods

### Mice

Male C57BL/6 mice, aged 4–8 weeks, were purchased from Shanghai SLAC Laboratory Animal Company and maintained on a standard chow diet with a 12 h light–dark cycle. One week after arrival, mice were randomly divided into different groups according to experiment. Mice were kept on a standard chow diet or switched to HFD for a predetermined number of weeks. At this time, mice were sacrificed and adipose tissues were harvested or the adipocytes from SVF and adipocyte fractions were separated; detailed procedures are described in the Supplemental Experimental Procedures section. All animal procedures were approved by the Animal Care Committee of Shanghai Jiaotong University School of Medicine.

### Cell culture and reagents

3T3-L1, C3H10T1/2 and 293T cell lines were purchased from the American Cell Type Culture Collection (ATCC, Manassas, VA, USA) and cultured routinely in Dulbecco's modified Eagle's medium (DMEM) (Gibco, 11995, Grand Island, NY, USA) containing 10% fetal bovine serum (Gibco, 10099-141, Australia); detailed procedures are described in the Supplementary Experimental Procedures section.

### Lentivirus transduction

A lentivirus containing the Birc5 (Survivin) expression vector was ordered from the Shanghai Genepharma Corporation. The virus was used at a multiplicity of infection of 20 to infect 3T3-L1 preadipocytes, and the efficiency of infection was determined by the number of green-fluorescent-protein-positive cells after 48 h. GFP lentivirus was used as a control. Cells were passaged and differentiated into mature adipocytes for subsequent experiments. Overexpression of Birc5 was assessed by qRT-PCR and western blot analysis.

### Transcriptome analysis for 3T3-L1 adipocytes

3T3-L1 preadipocytes infected or not infected with Birc5 lentiviral vectors were cultured and differentiated as described in the previous section. On post-differentiation day 8, cells were treated with 50 ng/ml TNF*α* for 24 h; detailed procedures are described in the Supplementary Experimental Procedures section. Then paired-end libraries were synthesized using the TruSeq RNA Sample Preparation Kit (Illumina, RS-122-2001, SFO, CA, USA) following the supplied guidelines. Library construction and Illumina sequencing were performed at the Shanghai Biotechnology Corporation. High-quality reads that passed the Illumina quality filters were kept for sequence analysis and bioinformatic data analysis was performed by the Shanghai Novel Bioinformatics Company.

### RNA sequencing mapping and bioinformatic data analysis

Before read mapping, clean reads were obtained from the raw reads by filtering out rRNA reads, sequencing adapters, short-fragment reads and other low-quality reads. Clean reads after filtering were aligned to the mouse genome (GRCm38.83) by Tophat v 2.0.9.^33^ The differentially expressed genes were identified by EBSeq algorithms. Genes were considered significantly differentially expressed under the following criteria: (1) fold change>1.5, and (2) FDR<0.05.^34^

To identify potential functions of Survivin in adipocytes, RNA sequencing analysis of DEGs was performed using Serious cluster analysis, GO pathway analysis, GO tree analysis and Co-expression networks, and the detailed processes are described in the Supplementary Experimental Procedures section.

### Microarray

Microarray data showing gene expression changes in epididymal fat of HFD mice or ob/ob mice were reanalyzed here, which are available at the NCBI GEO database (http://www.ncbi.nlm.nih.gov/geo/) and can be accessed at GEO (GSE27017).

### Cellular triglyceride determination

For basal lipolysis measurements, cellular triglyceride content was measured using commercial kits (Biovision, K622-100, Mountain View, CA, USA) according to the manufacturer's instructions. Results were corrected for cellular proteins.

### Lipolysis measurement

Mature 3T3-L1 adipocytes were first treated with 1 *μ*M isoproterenol or 10 ng/ml TNF*α* in serum-free DMEM containing 0.2% BSA for 24 h, then incubated with serum-free DMEM containing 0.2% BSA for 3 h. Glycerol content in the incubation medium was used as an index for lipolysis and measured using GPO-Trinder reagent (Sigma, FG0100, St. Louis, MO, USA) based on the manufacturer's instructions. Results were corrected for cellular proteins.

### cAMP measurement

Cellular cAMP was measured using a cAMP enzyme immunoassay kit (Sigma, CA200, St. Louis, MO, USA), To measure cAMP levels, the differentiated 3T3-L1 adipocytes in 24-cell plates were incubated in 0.2% BSA for 6 h and subjected to 1 *μ*M ISO stimulation for another 1 h. In order to prevent the degradation of cAMP, ISO group was also treated with 200 *μ*M IBMX along with ISO stimulation. Then cells were immediately lysed and the cellular cAMP were measured according to the manufacturer's instructions. Finally, cAMP levels were calculated based on the manufacturer's instructions and corrected by celluar protein level.

### Quantitative real-time PCR analysis

Total RNA was isolated using the Trizol reagent (Invitrogen, 15596018, Carlsbad, CA, USA) and reverse transcription was performed using an iScript cDNA synthesis kit (Bio-Rad, 170-8891, Hercules, CA, USA) according to the manufacturer's instructions. qRT-PCR analysis analysis was carried out using SYBR Premix Ex Taq (Takara, RR820A, Japan) in a LightCycler480 PCR system (Roche, Germany). The sequences of primers are provided in Supplementary Table 1.

### Western blot analysis

Proteins in cells and adipose tissues were extracted with RIPA buffer (Sigma, R0278, St. Louis, MO, USA), separated by 15% SDS-PAGE, and transferred to nitrocellulose membranes (Whatman, 10402495, Florham Park, NJ, USA). Membranes were incubated with primary antibodies overnight at 4 °C. The membranes were incubated with horseradish peroxidase-conjugated secondary antibodies for an hour at room temperature and developed with the ECL Prime Western Blotting Detection Reagent (GE Healthcare, RPN2232, Piscataway, NJ, USA) using Image Quant LAS4000 Imaging Systems (GE Healthcare, Pittsburgh, PA, USA). Detailed information for these antibodies is described in the Supplementary Experimental Procedures section.

### *In vitro* co-immunoprecipitation

293T cells with ectopic Survivin expression were transfected with the flag-Fsp27 plasmid (provided by Prof Peng Li) according to the indicating constructs and were then harvested 48 h later. Cells were resuspended in lysis buffer (50 mM Tris–HCl pH 7.4, 150 mM NaCl, 1 mM EDTA, 1% Triton X-100) containing protease and phosphatase inhibitors. The lysates were immunoprecipitated with flag beads (Sigma, A2220, St. Louis, MO, USA) overnight at 4 °C and then immunoblotted with anti-flag (Cell Signaling, 2368, Danvers, MA, USA).

### Statistical analysis

The results are presented as means±S.E.M. for three independent experiments. Statistical differences were determined by two-tailed Student's *t*-test or one-way ANOVA. *P*-values<0.05 were considered significant.

## Figures and Tables

**Figure 6:**
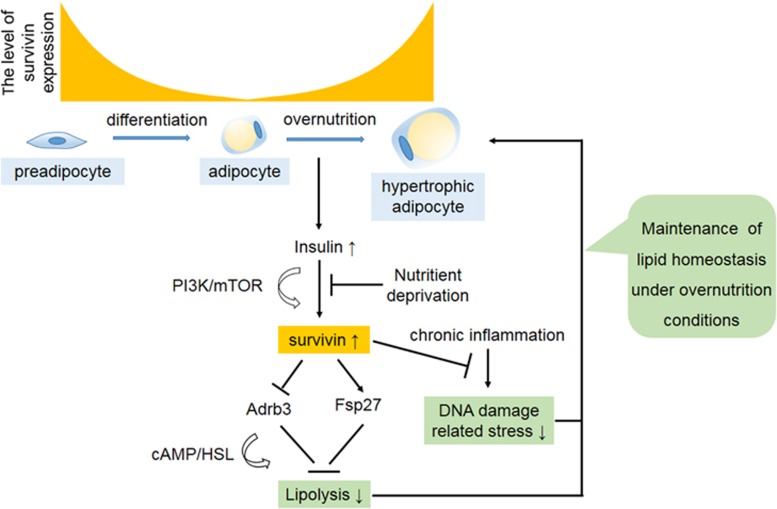
A working model summarizing the role of Survivin in adipocyte homeostasis. During adipocyte differentiation, Survivin expression is gradually decreased and almost undetectable in mature adipocytes. Upon overnutrition condition, adipocytes are able to expression the Survivin gene via elevated insulin levels through the PI3K/mTOR pathway. Re-expressed Survivin inhibits isoproterenol-stimulated adipocyte lipolysis via decreased Adrb3 content, as well as increased Fsp27 expression. Survivin also attenuates TNF*α*-induced lipolysis and DNA damage-related stress responses. Taken together, the current findings suggest that Survivin may facilitate adipocyte maintenance in response to obesity-induced adipocyte stress
