# Supplementary material for: Enhanced expression of Survivin has distinct roles in adipocyte homeostasis
Source: Cell Death Dis. 2017 Jan 5;8(1):e2533–. doi: 10.1038/cddis.2016.439 (PMC5386358; doi:10.1038/cddis.2016.439)
Supplement: Supplementary Figure and Table Legends [file cddis2016439x4.doc]

**Enhanced expression of Survivin plays distinct roles in adipocyte homeostasis**

Running title: Critical role of Survivin in adipocyte homeostasis

Liping Ju^1*^, Xiaoyan Zhang^2*^, Yujie Deng ^3^, Junfeng Han^1^, Jian Yang^2^, Shuqin Chen^1^, Qichen Fang^1^, Ying Yang^1^, Weiping Jia^1^

**Supplementary Table S1:**

Primers for quantitative real-time PCR analysis in mice

| Genes | Forward | Reverse |
| --- | --- | --- |
| Birc5(Survivin) | GAGGCTGGCTTCATCCACTG | ATGCTCCTCTATCGGGTTGTC |
| Adrb3 | TCTCTGGCTTTGTGGTCGGA | GTTGGTTATGGTCTGTAGTCTCG |
| Tshr | AGAACTGATCGCAAAAGACACC | CCGGATACTGCTCTCATTACAC |
| Adcyap1r1 | CTGCGTGCAGAAATGCTACTG | AGCCGTAGAGTAATGGTGGATAG |
| Pde1b | AGTTCCGAAGCATCGTGCAT | CTTGAGACAGTTGTGGACTGC |
| Adcy5 | CTTGGGGAGAAGCCGATTCC | ACCGCTTAGTGGAGGGTCT |
| Cidea | TGACATTCATGGGATTGCAGAC | CATGGTTTGAAACTCGAAAAGGG |
| Cidec | ATGGACTACGCCATGAAGTCT | CGGTGCTAACACGACAGGG |
| Plin1 | CTGTGTGCAATGCCTATGAGA | CTGGAGGGTATTGAAGAGCCG |
| Neil3 | TCCCTGGCTGATGTCGCTA | AGCTCCTTCCCTAAGGTTTCC |
| Rad51 | GTCCACAGCCTATTTCACGGT | ACAGCCTCCACTGTATGGTAAC |
| 2810417H13Rik | ACCAAAGCAAACTACGTTCCA | TTTTCCCGACGAACTTGAAGAA |
| Pole | CTCAGCACTCAAGCGTCTGG | GCCACCTTAAATCTGCTTCCAT |
| Top2a | TGCTCCGCCCAGATACCTAC | TGGGTCCCTTTGTTTGTTATCAG |
| Exo1 | ATGGGGATTCAAGGGTTACTTCA | AGCCAACAGTAGGTATCCACAG |
| Plk1 | CCCGCTGGCGAAAGAAATTC | CATTTGGCGAAGCCTCCTTTA |
| Pparg | TCGCTGATGCACTGCCTATG | GAGAGGTCCACAGAGCTGATT |
| Cebpa | CAAGAACAGCAACGAGTACCG | GTCACTGGTCAACTCCAGCAC |
| Ppargc1a | TATGGAGTGACATAGAGTGTGCT | CCACTTCAATCCACCCAGAAAG |
| 36b4 | AAGCGCGTCCTGGCATTGTCT | CCGCAGGGGCAGCAGTGGT |

**Figure S1 related to Figure 3**

**Fig S1 Overexpress survivin in 3T3-L1 adipocytes has not a significant effect on cell differentiation and glycerol release**

3T3-L1 cells were infected with Birc5 lentivirus to overexpress Survivin. (A) 8 post differentiation adipocytes were observed by microscope to evaluate the lipid droplets pattern. Original magnification, ×100. (B) The protein levels of PPARγ, C/EBPα and Survivin were monitored by immunoblotting of cell lysates which were extracted at indicated time during adipocyte differentiation. (C) Cellular triglyceride under basic condition between control and Survivin overexpression group. (D) Data analysis of RNA sequencing on genes related with lipid metabolism, such as adipocyte differentiation, fatty acid β-oxidation, lipogenesis, lipase, lipid droplet envelope protein and lipolysis associated protein. (E) Adipocyte differentiation related protein were tested by qRT-PCR in 3T3-L1 adipocytes to validate the results of RNA sequencing. ***: p <0.001.

**Figure S2 related to Figure 5**

**Fig S2 RNA sequencing analysis of survivin function in adipocytes with TNFα treatment**

The mRNA expression differences were detected by RNA sequencing analysis. DEGs were chose to do a deep analysis. (A) Series cluster analysis was introduced to discover the expression trend of DEGs from control, TNFα and Survivin overexpression+TNFα group. Then profile 5 and profile 2 of series cluster analysis were merged and DEGs were used to do a deep analysis (B-D). (B) DEGs were used to do a significant gene ontology analysis. *P* value < 0.05 for all significant GO terms. (C) Go tree analysis of DEGs based on Gene Ontology Directed Acyclic Graph. *P* value < 0.01 for all significant GO terms. In Survivin overexpressed 3T3-L1 adipocyes, mRNA level (D) and protein content (E) of Survivin were tested after 6 days of TNFα treatment. ***: p <0.001.
